# Environmental Regulation, Foreign Direct Investment and Green Technological Progress—Evidence from Chinese Manufacturing Industries

**DOI:** 10.3390/ijerph15020221

**Published:** 2018-01-29

**Authors:** Jiangfeng Hu, Zhao Wang, Yuehan Lian, Qinghua Huang

**Affiliations:** School of Economics and Management, Southwest University, Chongqing 400715, China; leisure@email.swu.edu.cn (J.H.); wz777@163.com (Z.W.); hqh@swu.edu.cn (Q.H.)

**Keywords:** environmental regulation, FDI, green technological progress, spillover effect

## Abstract

This study examines the spillover effects of foreign direct investment (FDI) on green technology progress rate (as measured by the green total factor productivity). The analysis utilizes two measures of FDI, labor-based FDI and capital-based FDI, and separately investigates four sets of industry classifications—high/low discharge regulation and high/low emission standard regulation. The results indicate that in the low discharge regulation and low emission standard regulation industry, labor-based FDI has a significant negative spillover effect, and capital-based FDI has a significant positive spillover effect. However, in the high-intensity environmental regulation industry, the negative influence of labor-based FDI is completely restrained, and capital-based FDI continues to play a significant positive green technological spillover effects. These findings have clear policy implications: the government should be gradually reducing the labor-based FDI inflow or increasing stringency of environmental regulation in order to reduce or eliminate the negative spillover effect of the labor-based FDI.

## 1. Introduction

It is generally believed that developing countries reverse the situation that lacking of technological innovation in primary stage of development through utilizing the technology spillover effect of FDI [1]. Therefore, for a long time, China’s policy makers have always seen foreign funded enterprises as representative of high-tech enterprises, and make use of local resources in exchange for advanced technology in production and management to achieve economic growth. However, does FDI really bring beneficial technological spillover effect to host countries? Yet another, more serious environmental problem has emerged before the crucial question has been answered explicitly. The PM_2.5_ concentration in Beijing during 2013 significantly exceeded the health standard suggested by the World Health Organization (WHO) [2,3,4], and has caused the total economic losses of 246.26 billion Yuan (approximately 1.1% of the national GDP) during 2007 [5]. Not only that, the serious air pollution in China caused significant public health impacts [6,7]. The Global Burden of Disease (GBD) study undertaken by Institute for Health Metrics and Evaluation and World Health Organization (WHO) linked over 3.2 million premature deaths to PM pollution in 2010, and roughly one-third (1.23 million) of the world’s estimated PM pollution-related premature deaths were in China [8]. Given China’s relatively lax environmental policy, it has raised concerns among academics about whether the developing countries that led by China have become a “Pollution Haven” for multinational companies (MNCs). The purpose of this article is not the simple verification on FDI technology spillover effect and environmental effect. Rather, we attempt to explore whether environmental regulation as a kind of non-market approach can promote the technological spillover effect of FDI and environmental quality under the background of the increasing environmental protection pressure in China.

In recent years, it is not groundless that FDI will deteriorate the ecological environment of host countries, the argument is as follows. Firstly, the international division of labor is unceasing deepening which is supported by the global value chain. According to factor endowment theory, developed countries have the move to shift the pollution to developing countries because of the comparative advantage in sufficient labor force and lax environmental regulation [9,10,11,12,13]. Although this process has boosted the rapid growth of China’s economy, it has also led to serious “trade-induced” environmental pollution [14], which results in the increasingly serious pollution in China. Secondly, the local government who is order to obtain advantages in regional economy and politics will have sufficient incentives to reduce environmental standards, which called “race to the bottom” behavior [15]. This behavior will attract more foreign capital and other liquidity factors [16], so that developing countries become transnational corporations’ “Pollution Havens” [17]. This is the commonly studied “Pollution Haven Hypothesis (PHH)”. However, some scholars have argued that a large-scale entrance of FDI does not necessarily deteriorate the environmental quality in host countries. On the contrary, investment of the MNCs in host countries can bring more environmental friendly production standards and technologies, and have a positive impact on host countries’ environmental protection through the “demonstration effects”, this argument is known as a “Pollution Halo Hypothesis” [18]. It attracted the attention of many scholars once presented. Zugravu-Soilita [19] insisted that these foreign firms, although to some extent guided in their location choice by the “Pollution Haven Hypothesis” and merely complying with less severe regulations in the host country, are often found to be more environmentally efficient than local firms. Moreover, foreign companies might desire to use less polluting technologies are order to avoid backlash from constituencies of their home countries [20]. In a simple theoretical model, Elliott and Zhou [21] found support to the “Pollution Halo Hypothesis” when firms are assumed to be cleaner, which is usually characteristic of FDI from developed towards developing countries. 

The two conflicting viewpoints described above indicate that FDI can bring a green technological spillover effect of improving economic growth in the long run accompanied with an improved environmental performance for the host countries, only when the degree of technological cleanliness selected is closely related to the stringency of environmental regulation of the host countries, in other words, the spillover effect of FDI does not occur automatically. More and more evidences show that increasing stringency of environmental regulation is not hinder the entrance of FDI, but on the contrary, may extrude FDI of high cleanliness from high-polluting domestic firms [21], thus contributing to developing countries to achieve green development. Numerous studies have shown that, strict environmental regulation not only prompted the government to adjust the structure of foreign investment and improve the environment threshold of foreign investment, also filtered the FDI, which means giving priority to introduce FDI that is beneficial to environmental protection and technology upgrading, crowding out the FDI in pollution intensive industry. Dean and Lovely et al. [10] separately confirmed that the strict environmental regulation significantly inhibited the entrance of FDI which has low technology-intense, high pollution level and low R&D intensity through their studies, and had no significant effect to FDI in the clean type of high-tech industry. Tang [22] first noticed that significant variation exists in the process of environmental regulation influencing FDI which has different entry motivation, environmental regulation has a significant negative impact on vertical FDI, but has less effect on the horizontal FDI, and pointed out that the reason why most empirical studies related to “Pollution Haven Hypothesis” have differences is because they didn’t consider the heterogeneity of FDI.

In general speaking, the existing studies mostly emphasize the influence of environmental regulation on the choice of foreign investment entrance, even a few scholars studied the relationship between environmental regulation and FDI technology spillover effect, but it did not show whether FDI technology spillover effect is helpful for the improvement of the environment quality. According to the Acemoglu and Aghion et al. [23], technology can be classified as “dirty” technology and “clean” technology, when there is guidance of environmental regulation, technical progress is no longer just a simple increase in technical level, but has a certain bias. Chung and Fare [24] put forword a method of directional distance function, when measured the TFP of the Swedish pulp mill. This method takes pollution emission as unexpected. Then builds a Malmquist-Luenberger index based on on this basis. Since then, the method of measuring green total factor productivity has been widely used. But when there is an excessive input or insufficient output, which is if exist the non-zero relaxation of input or output (Slack), the radial type of DEA efficiency measurement will overestimate the efficiency of the object. Therefore, Färe and Grosskopf [25], Fukuyama and Weber [26] considered [27,28], and based on that developed a more general type of directional distance function based on non-radial SBM. Molinos-Senante and Maziotis et al. [29] measured the green total factor productivity of the UK and Welsh water companies according to the SBM function, which were decomposed by the Malmquist-Luenberger index and the Luenberger index respectively, and it is considered that the former overestimated the change of productivity. To make up for the lack of previous research, we based on SBM model and Luenberger index to measure the rate of green technological progress in 30 sectors of China industry from 2003 to 2010, and proceeded empirical research on FDI technology spillover effect under the situation that fixed intensity of environmental regulation and variable intensity of environmental regulation.

The paper unfolds as follows: Section 2 is based on the SBM directional distance function and the Luenberger productivity index to measure the efficiency of China’s manufacturing green technology progress. Section 3 sets the indexes and classifies the manufacturing industry according to the strength of the two kinds of environmental regulation tools, and bases on our questions set two kinds of econometric models. Section 4 answers the question from the first part through analyzing the empirical results. Section 4 summarizes the whole paper and discusses the policy implications of the conclusions.

## 2. The Measure of the Manufacturing Green Technology Progress

The focous of this paper is that the measurement of green technology progress of manufacturing industry. We use the SBM directional distance function to measure the efficiency of 30 manufacturing industries and the Luenberger index to decompose the manufacturing green technology progress.

### 2.1. Methods

#### 2.1.1. Production Possibility Set Considering Environmental Factors

In this paper, each industry is used as a decision making unit (DMU) to construct the production frontier, assuming that each DMU uses N input, $x_{\mathrm{jn}}\left(n=1,\;\ldots ,\;N\right)\in R^{+},$ j is the industry, obtain M expected outputs, ${\mathrm{yg}}_{\mathrm{jm}}\left(m=1,\;\ldots ,\;M\right)\in R^{+}$, P non-anticipated outputs, ${\mathrm{yb}}_{\mathrm{jp}}\left(p=1,\;\ldots ,\;P\right)\in R^{+}$, DEA can be used to express the production frontier of non-expected outputs as follows:

(1) $$P^{t}(x^{t})=\{\left({\mathrm{yg}}^{t},{\mathrm{yb}}^{t}\right):\overset{J}{\underset{j=1}{\sum }}\lambda_{j}^{t}{\mathrm{yg}}_{\mathrm{jm}}^{t}\geq {\mathrm{yg}}_{\mathrm{jm}}^{t},\;\forall m;\;\overset{J}{\underset{j=1}{\sum }}\lambda_{j}^{t}{\mathrm{yb}}_{\mathrm{jp}}^{t}\geq {\mathrm{yb}}_{\mathrm{jp}}^{t},\;\forall p;\;\overset{J}{\underset{j=1}{\sum }}\lambda_{j}^{t}x_{\mathrm{jn}}^{t}\geq x_{\mathrm{jn}}^{t},\;\forall n;\;\overset{J}{\underset{j=1}{\sum }}\lambda_{j}^{t}=1,\;\lambda_{j}^{t}\geq 0,\;\forall j\}$$

where ${x=(x}_{1},\;\ldots ,\;x_{N}),\;\mathrm{yg}=({\mathrm{yg}}_{1},\;\ldots ,\;{\mathrm{yg}}_{M}),\;\mathrm{yb}=({\mathrm{yb}}_{1},\;\ldots ,\;{\mathrm{yb}}_{P}),\;\lambda_{j}^{t}$ is the weight of the observed values for each cross section.

#### 2.1.2. SBM Directional Distance Function

We definite the directional distance function under the energy environment according to Fukuyama and Weber [26]:

(2) $$\vec{S_{V}^{t}}(x^{t,j^{\prime }},yg^{t,j^{\prime }},{\mathrm{yb}}^{t,j^{\prime }},g^{x},g^{yg},g^{yb})=\underset{s_{n}^{x},s_{m}^{\mathrm{yg}},s_{p}^{\mathrm{yb}}}{\max}\frac{\frac{1}{N}\sum_{n=1}^{N}\frac{s_{n}^{x}}{g_{n}^{x}}+\frac{1}{M+P}(\sum_{m=1}^{M}\frac{s_{m}^{yg}}{g_{m}^{yg}}+\sum_{p=1}^{P}\frac{s_{p}^{yb}}{g_{p}^{\mathrm{yb}}})}{2}\;s.t.\;\sum_{j=1}^{J}\lambda_{j}^{t}x_{\mathrm{jn}}^{t}+s_{n}^{x}=x_{\mathrm{jn}}^{t},\forall n;\sum_{j=1}^{J}\lambda_{j}^{t}{\mathrm{yg}}_{\mathrm{jm}}^{t}-s_{m}^{\mathrm{yg}}={\mathrm{yg}}_{\mathrm{jm}}^{t},\forall m;\sum_{j=1}^{J}\lambda_{j}^{t}{\mathrm{yb}}_{\mathrm{jp}}^{t}-s_{p}^{\mathrm{yb}}={\mathrm{yb}}_{\mathrm{jp}}^{t},\forall p;\;\overset{J}{\underset{j=1}{\sum }}\lambda_{j}^{t}={1,\;\lambda }_{j}^{t}\geq 0,\forall j;\;s_{n}^{x}\geq 0,\forall n;\;s_{m}^{\mathrm{yg}}\geq 0,\forall m;\;s_{p}^{\mathrm{yb}}\geq 0,\forall p$$

In the above equation, $\vec{S_{V}^{t}}$ is the directional distance function of variable returns to scale (VRS) $\sum_{j=1}^{J}\lambda_{j}^{t}=1,$ if take out $\sum_{j=1}^{J}\lambda_{j}^{t}=1$ is $\vec{S_{C}^{t}}$ the directional distance function of constant returns to scale (CRS); $\left(x^{{t,j}^{\prime }},{\mathrm{yg}}^{{t,j}^{\prime }},{\mathrm{yb}}^{{t,j}^{\prime }}),\;(g^{x},g^{\mathrm{yg}},g^{\mathrm{yb}})\;\mathrm{and}\;(s^{x},s^{\mathrm{yg}},s^{\mathrm{yb}}\right)$, separately represent the input and output vectors, direction vectors and slack vectors of j industry. 

#### 2.1.3. The Luenberger Productivity Indicator (LPI)

Since Färe and Grosskopf [30] developed the Malmquist productivity index, was which proposed by Caves and Christensen et al. [31], the Malmquist productivity index is widely applied to a variety of fields. Chung and Fare [24] extended it to the Malmquist–Luenberger (ML) index which contains environmental factors. Whether the M index or ML index is used, the measurement angle is under the hypothesis of minimizing cost or maximizing the profit, which means the measurement method is about input or output. Chambers and Fāure et al. [32] developed a new method Luenberger productivity index, which is used without choosing the measurement angle and considering both the reduction of investment and the increasing of output. It corresponds to the hypothesis of maximizing profit, and the situation of minimizing cost and maximizing profit could be considered. Therefore, the Luenberger productivity index is a generalization of M index and ML index [33]. 

The Luenberger total factor productivity (LTFP) indicators between t and t + 1 periods as follows Chambers and Fāure et al. [32]:

(3) $$P^{t}(x^{t})=\{\left({\mathrm{yg}}^{t},{\mathrm{yb}}^{t}\right):\overset{J}{\underset{j=1}{\sum }}\lambda_{j}^{t}{\mathrm{yg}}_{\mathrm{jm}}^{t}\geq {\mathrm{yg}}_{\mathrm{jm}}^{t},\;\forall m;\overset{J}{\underset{j=1}{\sum }}\lambda_{j}^{t}{\mathrm{yb}}_{\mathrm{jp}}^{t}\geq {\mathrm{yb}}_{\mathrm{jp}}^{t},\forall p;\overset{J}{\underset{j=1}{\sum }}\lambda_{j}^{t}x_{\mathrm{jn}}^{t}\geq x_{\mathrm{jn}}^{t},\forall n;\overset{J}{\underset{j=1}{\sum }}\lambda_{j}^{t}=1,\lambda_{j}^{t}\geq 0,\forall j\}$$

Further, the LTFP can be decomposed into two components: efficiency change (Effe) and technical change (Tech) [34].

(4) LTFP=Effe+Tech

(5) $${\mathrm{Effe}}_{t}^{t+1}=\vec{S_{c}^{t}}{(x}^{t},{\mathrm{yg}}^{t},{\mathrm{yb}}^{t},g)-\vec{S_{c}^{t+1}}(x^{t+1},{\mathrm{yg}}^{t+1},{\mathrm{yb}}^{t+1},g)$$

(6) $${\mathrm{Tech}}_{t}^{t+1}=\frac{1}{2}[\vec{S_{c}^{t+1}}{(x}^{t},{\mathrm{yg}}^{t},{\mathrm{yb}}^{t},g)-\vec{S_{c}^{t}}(x^{t},{\mathrm{yg}}^{t},{\mathrm{yb}}^{t},g)]+[\vec{S_{c}^{t+1}}(x^{t+1},{\mathrm{yg}}^{t+1},{\mathrm{yb}}^{t+1},g)-\vec{S_{c}^{t}}(x^{t+1},{\mathrm{yg}}^{t+1},{\mathrm{yb}}^{t+1},g)]$$

The LTFP and its components can be interpreted as follows: (i) an LTFP>0 means an improvement in the productivity; (ii) an LTFP<0 means worsening of the productivity; and (iii) an LTFP=0 means that the productivity has not changed. 

Although previous studies generally decomposed LTFP into changes in technical efficiency and scale efficiency, but considering the changes of the manufacturing green technology mainly showed on the total factor productivity change. This change may come from the foreign technology absorption capacity and good foreign production management system, and perform certainly as the introduction of new technology and technological innovation, so this article only considers LTFP as an index to measure the technological progress in manufacture.

### 2.2. Relevant Data Processing

Measure the industry green technological progress also needs to construct the correlative indexes of the expected output, unexpected output and factor inputs, and the data required from the National Bureau of Statistics of the People’s Republic of China [35].

Expected output: This paper sets the gross value of industrial output as expected output, and make 1990 for the base period, to deflate the gross value of industrial output in each year with various industries PPI.

Unexpected output: Academics generally view waste emissions as unexpected output. To measure the manufacturing green technology progress comprehensively, we select waste water, waste gas, solid waste as unexpected output.

Factor inputs: Capital stock, most of the existing research using the perpetual inventory method to measure the current capital stock, but this kind of method requires higher data quality. Considering the data availability and quality, we calculate the capital stock in current period; Labor capital investment, as the labor time of all industries is not available, in this paper, we utilize the average workers in China Industrial Economy Statistical Yearbook instead of the labor-based investment; as for total energy input, we choose industrial energy consumption as indicators of energy input.

### 2.3. The Measure Results and Analysis

According to the above theory method and index, we used the Matlab2015b software to estimate the change of the manufacturing LTFP that considers an unexpected output of waste water, waste gas and solid waste.

**Figure 1:**
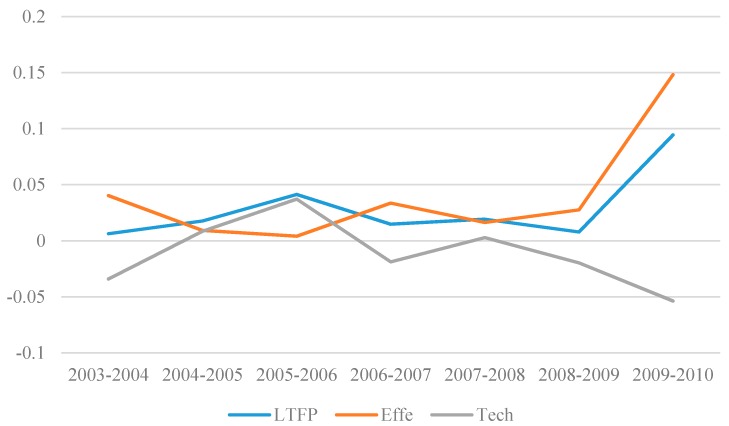
Evolution of the average values of the LTFP, Effe and Tech.

Figure 1 shows that the rate of China’s overall manufacturing green technology progress is changing positively, illustrating manufacture has not been locked as polluting industries in China. Specifically, LTFP is in accelerated phase in 2003–2006, growth rate increased from 0.627% to 4.14%, The engine of growth is mainly from Tech. 

It should be highlighted that the changes in technological use efficiency and technological progress have disintegrated during this period. The efficiency of technology utilization decreased from 4.03% to 0.41%, while −3.4% increased to 3.72%. During the years 2007 to 2009, the speed of China’s overall manufacturing green technology progress is slow, but the direction is still positive. The main reason is that the Tech declined significantly, and the insufficient contribution of Effe to the LTFP. There are two reasons for this drop. Firstly, influenced by the global financial crisis in 2008, the enterprises’ R&D fund dropped sharply and insufficient R&D investment in green technologies lead to a slow green technology progress. Secondly, the protection of the environment is the basic state policy of China, the upgrade of green manufacturing conforms to China’s long-term development goals. During this time, the enterprise which causes high pollution and high energy consumption should be shut down gradually, thus avoid the progress of manufacturing technology transfers to polluted technology. In the sample of this paper, the LTFP peaked in 2010 which is 9.45%. The main reason is that technological progress (Tech) showed a spurt of growth, rising from 2.76% in 2009 to 14.82% in 2010.

## 3. Variable Setting, Manufacturing Classification and Measurement Model

### 3.1. Variable Setting and Data Processing

#### 3.1.1. Core Independent Variable

Since this paper mainly focuses on the problem of the green technology spillover effect of FDI in the two situations of fixed and variable environmental regulation, the core independent variables mainly include environmental regulation and FDI:

Environmental regulation: The intensity of environmental regulation: considering the matching and synergies exist between the absorbed incentives of environmental technology and selection of environmental regulation tool, as well as complex policy environment in China, a simple use of several pollutants’ emissions or remove quantity to generalize China’s environmental regulation tools will be incomplete, and there may be a deviation in the results. On the ground of related theory in selection of the environmental regulation tools, tools are divided into “controlled” tools and “incentive” tools. The former is compulsive, mainly to achieve emission reduction in the command-control way, but will result in an increased cost and declining productivity [36]; the latter is to give enterprises preferential policy on clean research and development, such as subsidies on environmental protection technology research and development to provide continuous incentives in technical innovation and high efficiency of the enterprise, promoting enterprise technology progress towards a clean-type. Based on this, this paper constructs two kinds of environmental regulation indicators. Owing to reliable information of China’s pollution taxes and clean technology R&D subsidy rate is unavailable, we set the ratio of operation cost in waste-water treatment facilities and the main business income and the ratio of operation cost exhaust in gas treatment facilities and the main business income as two sub-indexes to measure the discharge regulation, the target layer is discharge regulation (UEx). We use the ratio of the waste-water’s standard amount and waste-water’s emissions and the ratio of the amount of SO_2_ removal and SO_2_ emissions as the two sub indicators of pollutant discharge standard regulation, target for emissions standards regulation (UEy). 

FDI: Evidences show that the impact of different types of FDI on the host country’s economy and environment is quite different [37]. Theoretical models of MNCs categorize FDI into different types according to its motivation and production model. Markusen and Venables [38] divides FDI into vertical FDI and horizontal FDI. MNCs engaging in the former outsource certain stages of production to host countries in order to take advantage of the relatively cheap factors of production there, and the latter is mainly to save trade costs and to produce and sell products in the host country [22]. Compared with horizontal FDI, vertical FDI has negative impact on the environment of host country. However, most of the existing research is based on data at the national or enterprise level and seldom distinguishes FDI types at the industry level. The academia community generally divides the industries into labor-intensive and capital-intensive industries based on the dependence of industry on labor capital and material capital. Based on the above reasons, this paper takes the ratio of the number of foreign employees and the number of employees in the manufacturing industry as labor-based FDI, and the ratio of the capital of foreign businessmen, Hong Kong, Macao and Taiwan businessmen to the paid-up capital of the manufacturing industry is taken as capital-based FDI, which is respectively expressed as FDI1 and FDIk.

#### 3.1.2. Control Variables

Pollution emissions intensity (NUE): Acemoglu and Aghion et al. [23] argued that the stronger the degree of pollution, the more possibly the current manufacturer’s technical features tend to polluted, namely technology progress has path dependence, pollution is always bad for green technology progress. We use waste water and solid waste discharge data to proceed linear standardization and weighted sum of squares, these are used to obtain the pollution emission intensity of each industry. R&D intensity (RD): it is well known that the more R&D investment is more conducive to technological progress. However, based on the research of Acemoglu and Aghion et al. [23], advances in technology have the dual characteristics of pollution and clean, only under environmental regulation intensity will technology progress towards a cleaner type. This paper adopts the ratio of industries’ R&D appropriation expenditure and investment in fixed assets to measure R&D intensity. Cost-profit ratio (RCP): refers to the ratio between profit and cost in a certain period. To a certain extent, it can reflect various industries’ economic benefits of the cost of production and devoted expense, but also can reflect the ability to reduce costs. Energy production efficiency (EP): the manufacturing industry needs to consume a large amount of resources during the production process, and these resources are often non-renewable. Energy productivity is the directly manifest to reflect the characteristics of the industry energy consumption and pollution emissions and the important symbol to measure industry clean production, based on the ratio of industry output value and industry energy consumption energy efficiency. Capital stock per capita (k): Capital stock per capita utilize the composition of industries input factors to reflect the height of the industries. This paper set the ratio of net value of fixed assets and the average industry workers to weigh Capital stock per capita.

#### 3.1.3. Data Description and Processing

The data of the related variables are derived from the National Bureau of Statistics of the People’s Republic of China [35]. Considering the diversity of environmental regulation tools and manufacturing consistency of statistical caliber, this article only selected data from 2003 to 2010 as the research sample. And that the eleventh Five-Year Plan for the first time use significantly reduction of pollution emissions as the binding forces of economic and social development, samples in this article just cover the period of before and after the eleventh Five-Year Plan, which can further reflect how FDI affects the efficiency of FDI in Chinese manufacturing green technology on the perspective of the environmental regulation intensity differentiation. To be able to improve the sample size while maintaining the data quality, this paper adopts the method of median cycle replenishing to deal with the missing data in the sample.

### 3.2. Manufacturing Classification

Considering the different characteristics in pollution level of manufacturing industries, the policy makers will base on these characteristics to design the different environmental regulation intensity in the manufacturing industry. In this paper, the 30 manufacturing industries respectively are divided into low/high discharge regulation industry and low/high emission standard regulation industry, according to the median of discharge regulation average strength $\sum_{t}^{n}{\mathrm{UE}}_{\mathrm{xjt}}/n$, and emission standard regulation average intensity $\sum_{t}^{n}{\mathrm{UE}}_{\mathrm{yjt}}/n$, here, j denotes industry. Table 1 shows the manufacturing classification results.

Table 1 shows that China’s manufacturing discharge regulation strength is weaker than emissions standards regulation strength generally, it also fit the environmental policy in China which mainly relies on administrative command to achieve the goal of emission reduction for a long time. Low intensity discharge regulation industry mainly is high and new technology industry, the industry pollution levels are relatively low, naturally don’t have too much expenditure in pollution abatement, while high intensity discharge regulation industry are mostly those of high pollution and high energy consumption industries, among them paper products discharge regulation strength is up to 0.64; pollution discharge standard regulation strength is mainly related to the amount of pollution emissions and pollutants standard, the less pollution emissions or the more contamination standard quantity means the higher pollution emission standard. The industries of higher pollution discharge standard regulation strength in Table 1 are the new and high technology industries, these industries’ standard pollution emissions are either little or very high, this also is consistent with analysis of the blow-down cost, and low pollution emission intensity is mainly traditional industries, these industries show a high emission or low pollutant discharge capacity.

### 3.3. Econometric Model

Based on the above index and subdivision of manufacturing industry, this paper builds two basic econometric models to test and compare the green technological spillover effect of FDI in fixed intensity of environmental regulation and variable intensity of environmental regulation. Among them, Equation (7) is used to test the effect of FDI on green technological progress in manufacture under the existing environmental regulation intensity; on the basis of the Equation (4) model, Equation (8) adds two variables of environmental regulation, which is used to examine the influence of FDI on the manufacturing green technology progress under the condition of applying additional environmental regulation.

(7) $${\mathrm{lnLTFP}}_{\mathrm{it}}=\alpha_{0}+\alpha_{1}{\mathrm{lnFDIk}}_{i,t}+\alpha_{2}{\mathrm{lnFDIl}}_{i,t}+\alpha_{3}{\mathrm{lnNUE}}_{i,t}+\alpha_{4}{\mathrm{lnRD}}_{i,t}+\alpha_{5}{\mathrm{lnRCP}}_{i,t}+\alpha_{6}{\mathrm{lnEP}}_{i,t}+\alpha_{7}{\mathrm{lnk}}_{i,t}+\mu_{i,t}$$

(8) $${\mathrm{lnLTFP}}_{\mathrm{it}}=\beta_{0}+\beta_{1}{\mathrm{lnFDIk}}_{i,t}+\beta_{2}{\mathrm{lnFDIl}}_{i,t}+\beta_{3}{\mathrm{UEx}}_{\mathrm{it}}+\beta_{3}{\mathrm{UEy}}_{\mathrm{it}}+\beta_{4}{\mathrm{lnNUE}}_{i,t}+\beta_{5}{\mathrm{lnRD}}_{i,t}+\beta_{6}{\mathrm{lnRCP}}_{i,t}+\beta_{7}{\mathrm{lnEP}}_{i,t}+\beta_{8}{\mathrm{lnk}}_{i,t}+\varepsilon_{i,t}$$

Among them, i is industry, t represents the time, $\varepsilon_{i,t}$ is the error term, explained variable is indexing green technological progress rate, that is ${\mathrm{lnLTFP}}_{\mathrm{it}}.$ In econometric model, it can be converted to cumulative growth index with setting 2003 as the base period. Because some of the values are negative or zero, this paper refers to the practice of scholars such as Jena and Managi [39], after adding 1 to all the values, and continue to multiply by year, then carry out the logarithmic transformation. 

In order to solve the endogeneity problem of common panel model estimation, this paper refers to the estimation method of Egger and Pfaermayr et al. [40], using industry and time double fixed effect model. The final model is as follows:

(9) $${\mathrm{lnLTFP}}_{\mathrm{it}}=\alpha_{0}+\alpha_{1}{\mathrm{lnFDIk}}_{i,t}+\alpha_{2}{\mathrm{lnFDIl}}_{i,t}+\alpha_{3}{\mathrm{lnNUE}}_{i,t}+\alpha_{4}{\mathrm{lnRD}}_{i,t}+\alpha_{5}{\mathrm{RCP}}_{i,t}+\alpha_{6}{\mathrm{lnEP}}_{i,t}+\alpha_{7}{\mathrm{lnk}}_{i,t}+\beta_{t}+\beta_{i}+\mu_{i,t}$$

(10) $${\mathrm{lnLTFP}}_{\mathrm{it}}=\beta_{0}+\beta_{1}{\mathrm{lnFDIk}}_{i,t}+\beta_{2}{\mathrm{lnFDIl}}_{i,t}+\beta_{3}{\mathrm{UEx}}_{\mathrm{it}}+\beta_{3}{\mathrm{UEy}}_{\mathrm{it}}+\beta_{4}{\mathrm{lnNUE}}_{i,t}+\beta_{5}{\mathrm{lnRD}}_{i,t}+\beta_{6}{\mathrm{RCP}}_{i,t}+\beta_{7}{\mathrm{lnEP}}_{i,t}+\beta_{8}{\mathrm{lnk}}_{i,t}+\beta_{t}+\beta_{i}+\varepsilon_{i,t}$$

Here, $\beta_{t}$ denotes control time fixed effect, $\beta_{i}$ denotes control industry fixed effect.

## 4. Empirical Results and Discussion

To test for different environmental regulation intensity (in low environmental regulation intensity and high intensity of environmental regulation), how the FDI influence the manufacturing green technology progress, this paper makes an empirical analysis of the four types of manufacturing according to the above-mentioned classification.

In the meantime, in order to ensure the robustness of the regression, we cluster the standard error at the industry level. Econometric model regression results are displayed in Table 2.

### 4.1. Analysis of Regression Results under Low Environmental Regulation Intensity

From the regression results, the labor-based FDI and capital-based FDI have differently influence the manufacturing green technology progress, among them, labor-based FDI represented labor intensive which always have a negative spillover effect in the manufacturing green technology progress of China, while capital-based FDI represented capital intensive on the contrary, it also explains why academia has different opinions on the role of FDI spillover effect and its direction.

Specifically, model (1) means in the situation which is low intensity discharge regulation and does not impose additional environmental regulation, when the influence of the labor-based FDI on the rate of China’s manufacturing green technology progress is significantly negative, the influence of the capital-based FDI shows a strong positive spillover effect. When additional sewage charges and emission standards are applied to the model (2), the green technological spillover of the labor-based FDI is negative. However, the coefficient dropped to −0.088; the coefficient of spillover effect in capital-based FDI is slightly reduced, from 0.312 to 0.28, but strengthen environmental standard regulation will reverse transmission force the manufacturing green technology progress of China. It makes up for the loss of FDI in positive externality, which are accord with the “Porter hypothesis” [41], that is appropriately strengthening environmental regulation will help enterprises to carry out green technological innovation. Models (3) and (4) represent regression results in the low intensity environmental standards. In model (3), two types of FDI influence the rate of the manufacturing green technology progress of the opposite direction. When further imposed additional environmental regulation, we get the model (4). Labor-based FDI still showed a significant negative impact, and the coefficient rose from −0.16 to −0.169, while the coefficient of capital-based FDI was only 0.166. Overall, FDI has a negative impact on China’s green technology progress. The main reason is that the motive of labor-based FDI enters into China is primarily for seeking cheap labor capital and avoid the tighter environmental regulation in their motherland, their investment is mostly located in the bottom of value chain in the industry and assembly links. The link has the least added value, heaviest pollution, lowest technical content in the process of production, it’s not conducive to the host country to promote the efficiency of the manufacturing green technology progress. In contrast, capital-based FDI is mainly involved in the host country’s industry in the form of capital, to obtain a high return on capital. During the process of industrialization in developing countries, capital intensive industries are unable to purchase high environmental performance of mechanical equipment due to the shortage of funds, the capital-based FDI will not only convey the needed funds to upgrade equipment, but also promote the technical level of the local companies in host countries through “demonstration effect” and “learning effect”.

It should be highlighted that in the model (4) the impact of the two types of environmental regulations on the rate of green technology progress has also been differentiated. Among them, the charge regulation hinders the progress of manufacturing green technology, the coefficient is −0.023, while the emission standard regulation has a positive impact, the coefficient is 0.025. This indicates that the charge regulation directly increases the cost of enterprise regulation, may squeeze out the productive investment of the enterprise, and is not conducive to the progress of green technology in the manufacturing industry. Relatively speaking, emission standards are too low, and the emission standard regulation is appropriately raised, and the environmental efficiency that it brings may exceed the economic loss caused by it.

### 4.2. Analysis of Regression Results under High Environmental Regulation Strength

In high intensity of environmental regulation models, the influence of environmental regulation on the spillover effect of different types of FDI also has differentiation. In high intensity of charge regulation models, the spillover effects of the two types of FDI are not significant at the 10% significance level. However, in the regulation of high-intensity emission standards, only capital-type FDI continues to exert a positive spillover effect.

It is important to note that the industry of high intensity discharge regulation in the model (5), the innovation direction of the enterprise has been deflected, and R&D has been transformed from the negative influence of green technology progress to positive promotion, although not significant at 10% significance level. According to Krysiak [42], when the environmental regulation achieves a certain intensity will force manufacturers to control the pollution generated from the technology in the R&D process of technology, in order to reduce environmental costs as much as possible, which make technical progress gradually transfer to clean technology. In model (5) and model (6), the negative spillover of labor-based FDI is completely restrained. This indicates that the low intensity of environmental regulation is the main reason for the negative spillover of labor FDI, which indicates that the “pollution haven” hypothesis is established in low intensity environmental regulation industry. However, the positive spillover effect of capital-based FDI becomes insignificant at the 10% significance level. Overhead expenses at the end, however, is a kind of burden outside the manufacturer routine operation, if the government rely too much on administrative commands to realize pollution reduction, it will do harm to green technological positive spillover effect of capital-based FDI. Compare to high intensity discharge regulation, in model (7) which is the regression results of high intensity emission standard regulation and model (8) which added the additional environmental regulation variables, the capital-based FDI always plays a positive green technological spillover effect on China’s manufacturing industry. Moreover, compared with other subgroups, capital-based FDI has a stronger spillover effect in the regulation of high-intensity emission standards, the coefficients reach 0.423 and 0.420 respectively. For a long time, China’s intellectual property protection (IPR) system is still not perfect relative to developed countries. Under the background of weak intensity environmental regulation, capital-based FDI will adopt the environmentally friendly technology, which consistent with the minimum level of environmental regulation in host countries in order to avoid the risk of technology exposure. As a result, when increasing the level of environmental standard regulation, capital-based FDI at the same time will take the corresponding green technology to further enhance the spillover effect of FDI on China’s manufacturing green technology innovation.

### 4.3. Further Explanatory Other Control Variables

In addition, this paper also analyzes the influence of other control variables on the green technology progress. The regression results show that: (1) Pollution emission intensity (lnNUE) in all models has shown a significant negative effect. It indicates that the higher pollution intensity of production technology, the easier it is for the technology to exhibit pollution bias, which detrimental to the development of green technology [23]. (2) Cost-profit ratio (lnRCP) is always beneficial to promote green technology progress of China’s manufacturing industry, but it is only in the low intensity environmental regulation industry. This indicates that the tightening of environmental regulation, while reducing pollution emissions, but need to reduce economic output as a price [43,44]. (3) The per capital stock (lnk) only played a significant positive role in the low emission standard regulation and high discharge regulation. This indicates that the effect of lnk on the green technological progress of manufacturing industry is not only influenced by the intensity of environmental regulation, but also due to the choice of environmental regulation tools. (4) Energy production efficiency (lnEP) has shown a significant positive effect in all models. According to Porter [41], Porter and Linde [45], pollution is a manifestation of wasteful and inefficient use of resources in the production process. The reduction of pollution tends to be synergistic with the improvement of resource utilization. Therefore, strict environmental regulation not only will not weaken the competitiveness of enterprises, it also helps to promote green technology innovations to compensate for “compliance costs”.

## 5. Conclusions

The spillover influence of FDI on the manufacturing green technology progress has both positive promotion and negative hindrance, which are affected by their own structure and the strength of environmental regulation. Specifically, in the low discharge regulation and low emission standard regulation industry, labor-based FDI has a significant negative spillover effect, and capital-based FDI has a significant positive spillover effect. However, in the high-intensity environmental regulation industry, the negative influence of labor-based FDI is completely restrained, and capital-based FDI continues to play a significant positive green technological spillover effects.

The labor-based FDI and capital-based FDI generated differentiation in the direction of green technological spillover, and have path dependence. In any intensity of environmental regulation and whether impose additional environmental regulation, labor-based FDI shows negative spillover effect from beginning to end, the capital-based FDI performs positive green technological spillover effect all the time. As a result, developing countries including China should set about from two aspects. On the one hand, making reasonable environment policy to inhibit adverse impacts of labor-intensive foreign investment on the environment. On the other hand, formulating reasonable industrial policy, increasing the intensity of open, attracting foreign capital into heavy industries which are capital-intensive, and raising the level of green technology in domestic enterprises.

The direction and magnitude of foreign direct investment’s green technological spillover effect is influenced by the strength and tool of environmental regulation. In the low intensity of environmental regulation industry, the labor-based FDI performs significant negative spillover effect, but the negative spillover effect can be eased through strengthening environmental regulation. Noteworthily, in the industries which have different strength of environmental regulation, the effect of two environmental regulation tools is obviously different. In the industry of low-intensity environmental regulation, the effect of discharge regulation promoting green technological spillover effect of FDI is relatively good, but in the industry of high-intensity environmental regulation, the effect of emission standard regulation is better than the discharge regulation. Therefore, when the host countries are making environmental policy, it should not only stipulate reasonable intensity of environmental regulation according to industry characteristics, but also apply flexibly in terms of the different effectiveness of the environmental regulation tools, which will suppress the negative spillover of FDI and further improve the positive green technological spillover effect of FDI.

## Figures and Tables

**Table 1:** Classification According to the Discharge Regulation.

**Low Discharge Regulation**	**Coefficients**	**High Discharge Regulation**	**Coefficients**
Stationery and sporting goods	0.0023	Textile and garments	0.0634
Electric apparatus	0.0073	Feather hairiness	0.0744
Crafts	0.0084	Recovery processing	0.0871
Plastic products	0.0136	Metal-ware	0.1083
Flexible unit	0.015	Food manufacturing	0.1487
Dedicated device	0.0173	Pharmaceutical manufacturing	0.1503
Transportation facilities	0.0207	Beverage processing	0.1769
Cabinet making	0.0209	Textile industry	0.2075
Electronic equipment	0.0224	Oil processing	0.2697
Tobacco	0.0241	Chemical industry	0.283
Print media	0.0294	Chemical fiber	0.3213
Wood processing	0.0411	Industry with gold	0.3539
Rubber products	0.046	Industry without gold	0.462
Agricultural byproducts processing	0.0584	Black processing	0.5179
Instruments and apparatus	0.0601	Paper products	0.6403
**Low Emission Standard Regulation**	**Coefficients**	**High Emission Standard Regulation**	**Coefficients**
Agricultural byproducts processing	0.1093	Flexible unit	0.4
Beverage processing	0.2097	Chemical fiber	0.4032
Food manufacturing	0.2135	Metal-ware	0.415
Stationery and sporting goods	0.2278	Pharmaceutical manufacturing	0.4165
Wood processing	0.2407	Dedicated device	0.4301
Plastic products	0.2583	Transportation facilities	0.4348
Feather hairiness	0.2786	Cabinet making	0.4504
Paper products	0.2935	Print media	0.4525
Industry without gold	0.3209	Black processing	0.4538
Tobacco	0.3281	Instruments and apparatus	0.4588
Recovery processing	0.3444	Textile and garments	0.4605
Chemical industry	0.378	Electric apparatus	0.4693
Crafts	0.3886	Rubber products	0.508
Electric apparatus	0.3897	Oil processing	0.564
Textile industry	0.3933	Industry with gold	0.7512

**Table 2:** Panel Model Regression Results.

Variables	Low Charge Regulation		Low Emission Standard Regulation		High Discharge Regulation		High Emission Standard Regulation	
	(1)	(2)	(3)	(4)	(5)	(6)	(7)	(8)
lnFDIl	−0.101 ***	−0.088 **	−0.160 ***	−0.169 ***	−0.006	0.085	−0.087	−0.101
	(−4.81)	(−3.30)	(−6.03)	(−8.86)	(−0.06)	(0.75)	(−0.40)	(−0.44)
lnFDIk	0.312 **	0.280 **	0.162 *	0.166 **	0.146	0.120	0.423 *	0.420 *
	(2.58)	(2.47)	(2.12)	(2.25)	(1.16)	(0.85)	(2.11)	(2.14)
lnUEx		−0.011		−0.023 ***		0.032 **		−0.010
		(−1.22)		(−4.26)		(2.44)		(−0.61)
lnUEy		0.034 *		0.025*		−0.043		0.041
		(2.00)		(2.10)		(−0.94)		(0.35)
lnNUE	−0.127 ***	−0.114 **	−0.122 **	−0.104 **	−0.063 **	−0.062 *	−0.180 **	−0.178 **
	(−3.07)	(−2.92)	(−2.39)	(−2.37)	(−2.33)	(−1.98)	(−4.02)	(−3.84)
lnRD	−0.022	−0.027 *	−0.014	−0.016	0.044	0.043	0.009	0.009
	(−1.61)	(−1.97)	(−0.63)	(−0.77)	(1.20)	(1.20)	(0.23)	(0.23)
RCP	2.934 **	2.246 **	2.522 **	2.033 **	0.274	0.292	0.548	0.530
	(4.13)	(3.08)	(3.54)	(3.89)	(0.29)	(0.32)	(0.58)	(0.55)
lnk	0.248	0.274	0.391 ***	0.447 ***	0.336 **	0.273 **	0.102	0.095
	(1.05)	(1.20)	(6.66)	(7.61)	(4.13)	(2.90)	(0.75)	(0.70)
lnEP	0.377 *	0.378 *	0.302 **	0.353 **	0.303 **	0.265 **	0.207 *	0.203 *
	(1.86)	(1.88)	(3.29)	(3.78)	(3.01)	(2.66)	(1.99)	(2.03)
Sample size	120	120	120	120	120	120	120	120
R^2^	0.640	0.660	0.527	0.564	0.306	0.334	0.425	0.428
Fe or RE	FE	FE	FE	FE	FE	RE	FE	FE

Note: * for 10% level significant, ** for 5% level significant, *** for 1% level significant. Data sources: according to Stata 12.0 calculation results.
